# Th17/Treg Imbalance in Chronic Obstructive Pulmonary Disease: Clinical and Experimental Evidence

**DOI:** 10.3389/fimmu.2021.804919

**Published:** 2021-12-09

**Authors:** Juliana Dias Lourenço, Juliana Tiyaki Ito, Milton de Arruda Martins, Iolanda de Fátima Lopes Calvo Tibério, Fernanda Degobbi Tenorio Quirino dos Santos Lopes

**Affiliations:** Laboratory of Experimental Therapeutics (LIM-20), Department of Clinical Medicine, School of Medicine, University of Sao Paulo, Sao Paulo, Brazil

**Keywords:** Th17 cells (Th17), regulatory T cell (Treg), Th17/Treg imbalance, COPD, immune response

## Abstract

The imbalance between pro- and anti-inflammatory immune responses mediated by Th17 and Treg cells is deeply involved in the development and progression of inflammation in chronic obstructive pulmonary disease (COPD). Several clinical and experimental studies have described the Th17/Treg imbalance in COPD progression. Due to its importance, many studies have also evaluated the effect of different treatments targeting Th17/Treg cells. However, discrepant results have been observed among different lung compartments, different COPD stages or local and systemic markers. Thus, the data must be carefully examined. In this context, this review explores and summarizes the recent outcomes of Th17/Treg imbalance in COPD development and progression in clinical, experimental and *in vitro* studies.

## 1 Introduction

Chronic obstructive pulmonary disease (COPD) is an inflammatory disease characterized by airway and/or alveolar abnormalities that lead to persistent airflow limitations and respiratory symptoms (1). According to the World Health Organization, COPD is currently the third leading cause of death, and it is predicted to remain so until 2030 (2). Although the primary cause of COPD is exposure to tobacco smoke, only approximately 25% of smokers develop the disease, which suggests that certain genetic, epigenetic, or host factors are involved in the amplified inflammatory response observed in COPD individuals (3).

COPD patients, especially when they have the disease in severe stages and during exacerbations, present systemic inflammation, which is associated with an accelerated decrease in lung function. This inflammation is characterized by increased circulating proinflammatory cytokines and chemokines, the levels of acute phase proteins, and abnormalities in circulating cells (4).

The inflammatory response in COPD involves both innate and adaptive immune responses (5, 6). T cell-mediated adaptive immunity is deeply involved in the regulation of airway inflammation during COPD development. During the developmental stage in the thymus, T lymphocytes differentiate into CD8+ T and CD4+ T cells. CD8+ T cells are the subtype of lymphocytes present in increased amounts in patients with COPD and they release proteolytic enzymes such as perforins and granzyme-B. These enzymes cause the death of structural cells by apoptosis or necrosis leading to the degradation of the extracellular matrix and remodelling, which results in the obstruction of small airways (6–8). In turn, CD4+ T cells can differentiate into different subtypes: T helper (Th)1, Th2, Th17 and regulatory T (Treg) cells (3, 5, 9). The imbalance between proinflammatory and anti-inflammatory immune responses mediated by the different subsets of Th cells, such as Th17 and Treg cells, plays a pivotal role in the maintenance of inflammation and disease progression (10, 11).

For many years, CD4+ Th1 lymphocytes have been considered key players in the COPD inflammation process due to the high expression of cytokines related to the Th1 response, such as TNF-α (tumour necrosis factor-α) during exacerbations and interferon-γ (IFN-γ), in emphysematous patients (12–14). However, over the last decade, evidence from clinical and experimental studies has highlighted the importance of Th17 cells in promoting and maintaining the inflammatory process in COPD, as well as the failure of Treg cells to control this inflammatory process.

Th17 cells are characterized by the release of IL-17A, IL-17F and IL-22, which are associated with COPD progression and the exacerbation of alveolar destruction (9, 11, 15, 16). They are found mainly in the bronchial mucosa and express the transcription factor *RORγt* as a specific marker (15, 17). In contrast, Treg cells are responsible for the regulation of immune responses by suppressing inflammation and autoimmunity through the release of anti-inflammatory cytokines, such as IL-10 and transforming growth factor-β (TGF-β) (5, 9). These suppressive functions are dependent on the expression of the transcription factor *Foxp3* (18). IL-10 is an interleukin produced by macrophages, dendritic cells, B cells, CD8+ T cells and several CD4+ T subtypes, and is mainly known for its anti-inflammatory effects (19–22). These cells are recognized as limiting the production of proinflammatory cytokines and chemokines such as IL-1β, -6, -12, -18, TNF-α, and MIP-1, among others. In addition, in the absence of IL-10, the IL-23 levels may increase, leading to their differentiation into Th17 cells (11, 20, 23, 24).

Although clinical and experimental studies have described the Th17/Treg imbalance in COPD progression (10, 16, 25–35), there are discrepant results when comparing these studies. Moreover, these discrepancies are also observed when analyses are performed in different lung compartments or when comparing samples from lungs to peripheral blood.

In this context, this review intends to explore and summarize the recent outcomes of Th17/Treg imbalance in COPD development and progression in clinical, experimental and *in vitro* studies.

## 2 Th17/Treg in Clinical Studies

### 2.1 Local Response

The discussions around the Th17/Treg imbalance in COPD gained strength with the studies of Lee et al. (2007) and Cosio et al. (2009) (6, 12). Lee and colleagues (12) proposed that a reduction in the number of Treg cells was associated with the progression of COPD, causing an imbalance between the pro- and anti-inflammatory responses. In this study, a reduction in *Foxp3* mRNA expression and IL-10 secretion was observed in the tissue of patients with COPD, suggesting a lower regulatory response in those patients.

From these observations, it has been proposed that exposure to cigarette smoke induces innate immune cells to secrete proteolytic enzymes, releasing fragments of elastin that can be recognized as autoantigens and initiate a process of autoimmunity mediated by T and B cells against self-elastin fragments. In addition, the authors proposed that Treg cells are essential to control this inflammatory response in an attempt to suppress the response to autoantigens. In accordance, Cosio et al. (2009) (6) proposed that the progression of COPD and the severity of this disease are determined both by the ability of dendritic cells to stimulate T cells and by the immunoregulatory actions dependent on Tregs. They suggested that a serious failure in this control of the inflammatory process mediated by Tregs results in advanced stages of COPD.

#### 2.1.1 Th17/Treg Immune Response Markers

Proinflammatory markers that characterize the immune response mediated by Th17 cells are usually increased in COPD patients. The IL-17A and IL-17F cytokines are predominantly released by Th17 cells, which are linked to neutrophilic inflammation, as IL-17 induces airway epithelial cells to release neutrophil chemotactic chemokines such as CXC-chemokine ligand 1 (CXCL1) and CXCL8, attracting neutrophils into the airways (36). Di Stefano et al. (2009) evaluated Th17-related cytokines (IL-17A, IL-22 and IL-23) in bronchial biopsies of patients with stable COPD (15). Although IL-17A is considered the main effector cytokine of Th17 cells, an increase in IL-17-positive cells was detected only in the bronchial submucosa of individuals with COPD and healthy smokers. The authors showed no significant difference in IL-17A-positive cells in the bronchial epithelium or in the gene expression of this interleukin between smokers with or without COPD or among different stages of COPD (15).

Zhang et al. (2013) evaluated the levels of IL-17 and IL-22 in the sputum of nonsmokers, healthy smokers and patients at 4 different COPD GOLD stages (16). The IL-17A and IL-22 levels were increased in COPD stages III and IV compared to the other groups, and sputum IL-17A levels in COPD patients were positively correlated with sputum neutrophils, enhancing the inflammatory process (16). In agreement with these results, Roos et al. (2015) observed increased IL-17A levels in the bronchoalveolar lavage fluid (BALF) of patients with COPD during acute exacerbations induced by *Haemophilus infuenzae* (NTHi) (37).

Regarding the anti-inflammatory process mediated by the Treg response, decreased numbers of Treg cells in the bronchial epithelium were reported in patients with severe and very severe COPD compared to those with mild and moderate COPD and healthy smokers (38). These results demonstrated that lower levels of Treg cells in the blood and lungs were observed only in severe COPD stages. In addition, Chu et al. (2011) demonstrated decreased Foxp3+ cells and gene and protein expression in tissue samples from moderate and severe COPD patients compared to healthy smokers and never smokers (29).

Contrasting results were observed when evaluating the Treg response in BALF. Smyth et al. (2007) observed an increase in the number of Treg+ cells in the BALF of COPD and healthy smokers compared with nonsmokers (39). However, the authors observed that those cells expressed lower levels of CD27+, a marker of more potent regulatory activity, which suggests weaker T regulatory activity in BALF than peripheral blood mononuclear cells (39). More recently, Ström et al. (2020) compared the proportion of Tregs with regulatory function (Foxp3+/CD4+CD25bright) among COPD smokers, healthy smokers, and control subjects (40). The authors also found that, despite no significant difference found among the groups, the proportions of Tregs with regulatory function were significantly lower in COPD subjects with a rapid decline in lung function than in those with a nonrapid decline (40).

Zheng et al. (2018) evaluated the Th17/Treg imbalance in tissue samples of COPD stage I and II patients and compared them to healthy smokers and controls (41). They observed a progressive increase in Th17 cells and a decrease in Treg cells in the COPD group, as measured by both immunohistochemistry and flow cytometry (41). In a previous study, our group demonstrated the Th17/Treg imbalance in tissue samples from COPD subjects (42). In this study, an increase in IL-17+ cells was observed in both the COPD and healthy smoker groups, whereas Treg (Foxp3+) and IL-10+ cell numbers were decreased in the small airways of obstructed smokers compared with healthy smokers and control subjects. In contrast, the authors also noted an increase in Treg cells in lymphoid tissues (42).

Similar results were observed by Plumb et al. (2009), in which the authors found increased Treg cells in the follicles of moderate COPD patients compared with those in the follicles of smokers and nonsmokers (43). Additionally, Isajevs et al. (2009) demonstrated an upregulation of Foxp3+ expression in the large airways of COPD patients compared to controls, although a downregulation was observed in the small airways (44).

#### 2.1.2 The Importance of Intracellular Signalling in Th17/Treg Differentiation

CD4+ T cell subset differentiation is dependent on antigen (Ag) nature, the type of antigen presenting cells (APCs), and the cytokines present in the microenvironment. Within the APCs, there are the dendritic cells (DCs), which are the unique one with capacity to activate T cells and initiate primary immune responses in lymph nodes (45). Therefore, DC derived signals can promote CD4+ T helper cell differentiation and CD8+ cytotoxicity (5). In COPD, Shan et al. (2009) showed that human lung DCs isolated from subjects with advanced emphysema could differentiate CD4+ T cells into distinct effector subsets (46). The authors demonstrated that total CD4+ T cells from PBMC that were cocultured with CD1a+ from lungs of subjects with advanced emphysema, but not control subjects, secreted higher amounts of IFN-γ and IL-17A. CD1a+ is a marker of Langerhans cells, a subtype of dendritic cell with antigen-presenting functions. Moreover, CD4+ T cells cultured with CD1a+ DCs from control, but not emphysema, differentiated into Treg cells, evidenced by an up-regulation of Foxp3 expression (46).

The cytokines present in the microenvironment are, however, the most important element on the CD4+ T cell subset differentiation. Cytokines are recognized by their cell surface receptors, which activate the Janus kinase–signal transducer and activator of transcription (JAK–STAT) pathway. JAKs are tyrosine kinases that bind to the cytoplasmic regions of type I and II cytokine receptors that, once activated, lead to the recruitment and phosphorylation of STAT proteins (47). Th17 differentiation is induced by the expression of *RORγt*, which is dependent on the activation of STAT3 in the presence of IL-6, IL-21 and IL-23. In contrast, STAT5 can promote Treg skewing by regulating *Foxp3* expression in the presence of TGF-β and IL-2 (47–49). Despite the importance of STAT proteins in regulating immunological responses, including Th17 and Treg skewing, only a few studies have evaluated them in COPD.

An increase in RORγt gene and protein expression was observed in a study conducted on tissue samples from patients with moderate and severe COPD who underwent pulmonary resection for peripheral carcinoma (29). Yew-Booth et al. (2015) demonstrated that stage III and IV COPD patients present with an increase in phosphorylated STAT3 protein in the lungs compared to nonobstructed smokers and never smokers, although no difference was observed in STAT5 expression (50). In a previous study by our group, *RORγt* and *STAT3* gene expression was also found to be increased in the lungs of mild and moderate COPD patients compared to those of healthy smokers (27). These findings were accompanied by increased *IL-6* and *TGF-β* gene expression and IL-6 levels, demonstrating early Th17 skewing. In this study, despite the increase in *Foxp3* gene expression in the lungs, no difference was observed in *STAT5* and *IL-10* gene expression or IL-10 levels (27).

Within the process of cellular homeostasis, there are also the important role played by the Suppressors of Cytokine Signalling (SOCS) proteins. They are small intracellular proteins that are responsible for inhibiting the activation of STATs, block their recruitment to the cytokine receptor or inhibit their phosphorylation by JAKs (51). Thus, SOCS proteins are an essential piece on the regulation of CD4+ T cells skewing into their subtypes. Springer et al. (2013) evaluated the gene expression of *SOCS3*, a well-defined STAT3 inhibitor and therefore affects Th17 skewing (52), in bronchial mucosa from COPD patients (53). The authors found that *SOCS3* expression was downregulated compared to that in nonsmoker control subjects, consistent with a Th17 response (53). In turn, SOCS1 can promote Th17 differentiation by regulating SOCS3 and inhibiting IFN-γ release (51). In addition, SOCS1 are related to the integrity and function of Treg cells, through the maintenance of Foxp3 expression (54). In patients with stage II COPD, decreased SOCS1 expression was observed both in the bronchial mucosa and in alveolar bronchoalveolar lavage macrophages (55).

It is important to consider the limitations and difficulties when studying some molecular and immunologic COPD aspects in humans, especially in lung tissue samples. Usually, COPD patients who present with constant exacerbations or who are in severe stages of the disease are not often submitted to procedures where lung tissue samples can be obtained. In contrast, healthy smokers or nonsmokers are usually undergo these procedures due to a confirmed tumour or a suspicion of neoplastic disease, which can be a limitation of some studies. Moreover, comparing tissue findings with those of BALF and peripheral blood can be hard to correlate because most studies do not obtain these samples from the same individual. Thus, the data must be carefully examined since the results from studies of one compartment may not be applicable to others.

### 2.2 Systemic Response

COPD patients who present with persistent systemic inflammation usually have worsening clinical outcomes, which negatively impacts their comorbidities, including an increase in the frequency of exacerbations and the risk of death (56). Whether systemic inflammation occurs as a result of the release of inflammatory mediators (“spill over”) from the lungs, where the inflammation begins, or whether it is the result of some comorbidity that then affects the lungs is still uncertain (4, 57, 58).

Many studies that have evaluated systemic Th17 response markers have revealed their increased levels in advanced COPD stages. Wang et al. (2015) evaluated peripheral blood samples from individuals who had never smoked, smokers without obstruction and individuals with moderate (stages II and III) and severe (stage IV) COPD (10). Patients with moderate and severe COPD had a higher frequency of Th17 cells, elevated levels of *RORγt* mRNA expression and increased serum levels of IL-17A, IL-6, IL-21, IL-22 and IL-23. The authors also showed a lower frequency of Treg cells, and decreased *Foxp3* mRNA expression and serum level of IL-10 in those patients. Additionally, it was demonstrated that the increase in the Th17/Treg ratio was negatively correlated with the worsening of lung function in those patients (10). In accordance, Silva et al. (2018) observed that patients with stage III COPD have more circulating IL-6 and less IL-10 than individuals with stage I and II disease (31).

We observed similar results evaluating both plasma and isolated total leucocytes from the peripheral blood of nonobstructive smokers (NOS), patients with COPD stages I and II, and COPD stages III and IV (27). Increased *STAT3*, *RORγt*, *IL-6* and *TGF-β* gene expression were observed in white blood cells from the COPD III and IV patients compared to those in stages I and II and NOS. Although *STAT5* was increased in the COPD III and IV group, there was a decrease in *Foxp3* expression and IL-10 levels in the COPD I and II and COPD III and IV groups, respectively, which was different from the tissue findings (27).

Contrasting results were shown by Vargas-Rojas et al. (2011). They demonstrated a progressive increase in both Th17 and Treg cell subsets in peripheral blood mononuclear cells (PBMCs) from COPD patients and smokers without COPD compared to healthy subjects (59). The Treg increase was attributed to long-term exposure to cigarette smoke but not airway obstruction. Additionally, the authors demonstrated a negative correlation between Th17 cells and FEV_1_ and FEV_1_/FVC values. In accordance, Xu et al. (2019) demonstrated a negative correlation between the percentages of circulating Th17 cells, which was found increased in COPD patients compared to smokers and never smokers, and FEV1% predicted values (60). Moreover, Zhang et al. (2013) found that serum levels of IL-17A progressively increased within COPD stages, although no difference was observed in the serum IL-10 levels between COPD patients and healthy smokers (16).

#### 2.2.1 Th17/Treg Imbalance in Stable and Exacerbated COPD Patients

Clinical studies have been performed to better understand the Th17/Treg imbalance during COPD exacerbations, and most of them showed increased levels of both Th17 and Treg response markers.

Li et al. (2014) observed an increased Treg cell proportion in the peripheral blood of both acute exacerbation and stable COPD individuals compared to healthy smokers and an increased Th17 cell proportion only in the exacerbated COPD group (30). However, the authors observed a negative correlation between Th17 and Treg responses in both acute exacerbations and stable COPD. In contrast, Jin et al. (2014) (28) demonstrated an increase in inflammatory (IL-17 and TNF-α) and anti-inflammatory (IL-10 and TGF-β) cytokines in patients with exacerbated COPD, in addition to an increase in Treg cells. They suggested that although the Treg/IL-17 ratio showed normal values, Treg cells were insufficient to suppress the increase in mediators associated with inflammation.

Moreover, Zheng et al. (2018) found a progressive increase in Th17 cells in PBMCs of healthy smokers, individuals with stable COPD and those with exacerbated COPD compared to healthy nonsmokers (41). The authors also demonstrated a progressive decrease in Treg cells in those groups, evidence of a Th17/Treg imbalance. These results were in accordance with the lung tissue findings observed by the authors in the same study. However, PBMC samples were not obtained from the same subjects from which the lung tissue samples were collected (41). In fact, only a few studies have evaluated samples from lungs and peripheral blood obtained from the same patient (27, 38), which can be a limiting factor when studying human samples. **Table 1** summarizes the main findings regarding the Th17/Treg imbalance in clinical studies, divided by inflammatory marker and by systemic and local responses.

**Table 1:** Summary of the main findings regarding Th17/Treg imbalance in clinical studies.

CLINICAL STUDIES		
Inflammatory markers	Pulmonary response	Systemic response
**Treg**	↓ lung tissue of COPD (II-IV) vs. Control (12)	↓ peripheral blood of COPD (II-III) vs. Healthy smokers and Control (10)
	↑ lung tissue of COPD (I-II) vs. Healthy Smokers (27)	↓ peripheral blood of COPD (I-II) vs. Healthy smokers (27)
	↓ BALF of COPD (II-III) vs. Healthy smokers (61)	↑ peripheral blood of COPD (II-IV) vs. Control (59)
	↓ lung tissue of COPD (II-III) vs. Healthy smokers and Control (29, 41)	↓ peripheral blood of COPD (III-IV) vs. COPD (I-II) (38)
	↓ bronchial epithelium of COPD (III-IV) vs. COPD (I-II) (38)	↓ peripheral blood of Exacerbated COPD vs. Stable COPD (30)
	↓ BALF of COPD (rapid decline in lung function) vs. COPD (nonrapid decline) (40)	↓ peripheral blood of Exacerbated COPD vs. Stable COPD, Healthy smokers and Control (41)
	↓ small airways of COPD (I-IIII) vs. Control (42, 44)	
	↑ large airways of COPD (II) vs. Control (44)	
	↑ BALF of COPD (I-III) and Healthy smokers vs. Control (39)	
	↑ lymphoid tissue of COPD (I-III) vs. Control (42, 43)	
**IL-10**	↓ lung tissue of COPD (II-IV) vs. Control (12)	↓ serum of COPD (II-III) vs. Control (10)
	↓ small and large airways of COPD (I-III) vs. Healthy smokers and Control (42)	↓ plasma of COPD (III-IV) vs. COPD (I-II) (27, 31)
		↑ serum of Exacerbated COPD vs. Stable COPD and Control (28)
**Th17**	↑ lung tissue of COPD (II-III) vs. Healthy smokers and Control (29, 41, 50)	↑ peripheral blood of COPD (II-III) vs. Healthy smokers and Control (10)
	↑ lung tissue of COPD (I-II) vs. Healthy smokers (27)	↑ peripheral blood of COPD (II-IV) vs. Healthy smokers and Control (59)
		↑ peripheral blood of COPD (III-IV) vs. COPD (I-II) and Healthy smokers (27)
		↑ peripheral blood of Exacerbated COPD vs. Stable COPD and Healthy smokers (30, 41)5
**IL-17**	↑ bronchial submucosa of COPD (I-III) and Healthy smokers vs. Control (15)	↑ serum of COPD (III-IV) vs. COPD (I-II) (16)
	↑ sputum of COPD (III-IV) vs. COPD (I-II) (16)	↑ serum of COPD (II-III) vs. Healthy smokers and Control (10)
	↑ small and large airways of COPD (I-III) vs. Healthy smokers and Control (42)	↑ peripheral blood of Exacerbated COPD vs. Stable COPD and Control (28)
	↑ sputum of COPD during acute exacerbations (37)	↑ serum of Exacerbated COPD vs. Stable COPD and Control (28)

Data are presented divided by inflammatory marker and by the evaluated compartment (pulmonary and systemic response). Treg, regulatory T cell; IL-10, interleukin-10; Th17, T helper-17, IL-17, interleukin-17; COPD, chronic obstructive pulmonary disease; vs., versus; BALF, bronchoalveolar lavage fluid; ↑: increased; ↓: decreased.

## 3 Th17/Treg in Experimental Models of COPD

### 3.1 Local and Systemic Responses

Considering the difficulties of obtaining lung samples from patients, experimental studies are useful for studying different lung compartments and systemic responses in the same individual. Thus, we described the Th17/Treg imbalance in experimental models of COPD for both local and systemic responses in a single topic. Nevertheless, findings obtained from animal models are described in **Table 2**, discriminated by each different compartment.

**Table 2:** Summary of the main findings regarding Th17/Treg imbalance in experimental studies.

EXPERIMENTAL STUDIES		
Inflammatory markers	Pulmonary response	Systemic response
**Treg**	↑ lung tissue of Sub-acute CS exposed mice vs. Control (32)	↑ peripheral blood of Sub-acute CS exposed mice vs. Control (32)
	↓ lung tissue of Chronic CS exposed mice vs. Control (32, 33)	↓ peripheral blood of Chronic CS exposed mice vs. Sub-acute CS exposed mice and Control (32)
	↓ peribronchovascular areas of Sub-acute CS exposed mice vs. Control (25)	
	↑ lung parenchyma of CS and LPS exposed mice vs. Control (35)	
	↓ lung tissue of CS and NTHi exposed mice vs. CS exposed mice (34)	
**IL-10**	↓ lung tissue of Chronic CS exposed mice vs. Sub-acute CS exposed mice and Control (33, 62)	↓ serum of Chronic CS exposed mice vs. Sub-acute CS exposed mice and Control (32)
	↓ lung tissue of Chronic CS and Sub-acute CS exposed mice vs. Control (33, 62)	↑ serum of Sub-acute CS exposed mice vs. Control (32)
	↓ peribronchovascular areas of Chronic and Sub-acute CS exposed mice vs. Control (25, 26)	
	↓ BALF of Chronic CS exposed mice vs. Sub-acute CS exposed mice and Control (32)	
	↑ BALF of Sub-acute CS exposed mice vs. Control (32)	
	↑ lung parenchyma of CS and LPS exposed mice vs. Control (35)	
**Th17**	↑ lung tissue of Chronic CS exposed mice vs. Sub-acute and Control (32, 33, 62)	↑ peripheral blood of Chronic CS exposed mice vs. Sub-acute CS exposed mice and Control (32)
	↑ lung tissue of Sub-acute CS exposed mice vs. Control (33, 53)	
	↑ lung tissue of CS and NTHi exposed mice vs. CS exposed mice and Control (34)	
**IL-17**	↑ BALF of Chronic CS exposed mice vs. Sub-acute CS exposed mice and Control (32, 62)	↑ serum of Chronic CS exposed mice vs. Sub-acute CS exposed mice and Control (32)
	↑ lung tissue and BALF of Chronic CS exposed mice vs. Control (62)	
	↑ lung tissue of Chronic and Sub-acute CS exposed mice vs. Control (33, 62)	
	↑ lung tissue of Chronic CS exposed mice vs. Sub-acute CS exposed mice and Control (33, 62)	
	↑ peribronchovascular areas of Chronic CS exposed mice vs. Control (25, 26)	
	↑ lung parenchyma of CS and LPS exposed mice vs. Control (35)	
	↑ lung tissue of CS and NTHi exposed mice vs. CS exposed mice and Control (34)	

Data are presented divided by inflammatory marker and by the evaluated compartment (pulmonary and systemic response). Treg, regulatory T cell; IL-10, interleukin-10; Th17, T helper-17, IL-17, interleukin-17; CS, cigarette smoke; vs., versus; BALF, bronchoalveolar lavage fluid; LPS, lipopolysaccharide; NTHi, Haemophilus influenzae; ↑: increased; ↓: decreased.

Despite the evidence of a Th17/Treg imbalance and the worsening of lung function in clinical studies, some pathophysiological mechanisms can only be evaluated using animal models. In animal models, it is possible to perform temporal evaluations, which are crucial to obtain and improve the understanding of pro- and anti-inflammatory immune response changes during COPD development, including at different time points during disease progression.

In a temporal analysis, Wang et al. (2012) evaluated the lung tissue, BALF and peripheral blood of mice exposed to cigarette smoke (CS) for 4 and 24 weeks (32). The authors showed that mice chronically exposed to CS presented an increased Th17 prevalence (CD4+IL-17+/CD4+ T cells) in lung tissue and peripheral blood and increased *RORγt* mRNA expression in the lungs compared with control and sub-acute CS exposed mice. Moreover, chronic exposure led to increased levels of Th17-related cytokines (IL-17A, IL-6, IL-23) in BALF and serum. In contrast, the Treg response was first increased in subacute CS exposure group compared to control mice and then it decreased in the chronic exposure group. Additionally, the Treg prevalence (CD4+CD25+ Foxp3+/CD4+ T cells) in both the lung tissue and peripheral blood and *Foxp3* mRNA expression in the lungs and IL-10 in serum and BALF were significantly decreased in mice chronically exposed to CS (32).

Duan et al. (2016) showed similar results in mice exposed to CS for 12 and 24 weeks (33). The authors observed increased frequencies of Th17 (CD4+IL-17+) cells, *RORγt* mRNA expression and IL-6, IL-17, and TGF-β1 levels in the lungs of both groups exposed to CS compared to the control group, with higher values in the 24-week group. These findings were accompanied by a progressive decrease in Treg (CD4+Foxp3+) frequencies and *Foxp3* and IL-10 expression in the CS-exposed groups. Additionally, the frequencies of Tregs were negatively correlated with Th17 cells (33). These findings were consistent with a previous study from the same group, in which it was demonstrated that Th17 cell, *RORγt* and *IL-17A* mRNA expression were increased in the lungs of CS-exposed mice, even after 12 weeks of smoke exposure cessation (63).

In a previous study, we showed in temporal analyses that mice exposed to CS for 1, 3 and 6 months presented an increase in IL-17+ cells in the peribroncovascular area after the sixth month of exposure (25). However, reductions in IL-10+, TGF-β+ and Treg cell numbers were observed since the initial events of COPD development and were associated with decreases in lung function. Although the numbers of Treg cells in mice exposed to CS returned to numbers similar to those in the control group in later stages, the reductions in IL-10+ and TGF-β+ cell numbers persisted until the end of the protocol, suggesting that despite the amount of Treg cells present in the lung tissue, there is an impairment of immunosuppressive activity (25).

Thus, in another study, we demonstrated decreases in intracellular signalling for Treg cell differentiation and IL-10 release in peribronchovascular areas of the lung that occurred prior to Th17 signalling in mice exposed for 3 and 6 months to CS (26). A decrease in STAT5+ and pSTAT5+ cells and IL-10 expression was demonstrated at the 3rd month of exposure, whereas Th17 skewing was detected only at the 6th month with increased STAT3+ and pSTAT3+ cell numbers and IL-17 and IL-6 levels. We also demonstrated an increase in SOCS1+ density from the 3rd month of exposure, concomitantly with a decrease in SOCS3+ density (26). In agreement, Zhou et al. (2015) evaluated the Th17 response in mice exposed to CS for 2, 8, 12 and 24 weeks (62). They observed increased IL-17 levels in the lungs since week 2 that persisted until week 24, increased Th17 cells (characterized as CD3+CD8-IL-17A+) in the lungs since week 8, and increased IL-17 levels in the BALF of groups exposed to CS for 12 and 24 weeks. Interestingly, *RORc* and *STAT3* mRNA expression was increased only in mice exposed to CS for 8 and 12 weeks, while increased *IL-6* mRNA was also observed, but not in the 24-week-exposed group (62). These findings corroborate Ruwanpura et al. (2014), who also demonstrated in mice that IL-6 induced STAT3 activation, leading to Th17 cell differentiation during the inflammatory process (64).

### 3.2 Animal Models of COPD Exacerbation

Th17/Treg imbalance was also demonstrated by our research group to lead to an exacerbation of the inflammatory process in an animal model of CS exposure associated with lipopolysaccharide challenge (35). We showed that mice exposed to CS and associated with LPS instillation showed an increase in the inflammatory mediators STAT3+, pSTAT3+ and IL-17+, as well as the anti-inflammatory mediators STAT5+, pSTAT5+ and Treg (Foxp3+). However, despite the increase in Treg cells, the decrease in IL-10+ cell density observed in the CS/LPS group suggests that the failure of cytokine release plays a fundamental role in exacerbation of the inflammatory response (35).

In another experimental model of COPD exacerbation, female mice were exposed to CS and then were administered *Haemophilus influenzae* (NTHi) (34). A progressive increase in IL-17 levels in the serum and IL-6 levels and RORγt gene and protein expression in the lungs of the CS, NTHi and CS+NTHi groups compared to the control group was found. However, *Foxp3* gene expression was decreased in the CS+NTHi group, although no differences were observed among the groups when analysing the Foxp3 protein levels using IHQ and WB. The authors concluded that NHTi infection impaired the anti-inflammatory Treg balance, which combined with the strong effect of Th17 cells in the lungs leads to a Th17/Treg imbalance and AECOPD development (34). In accordance, Roos et al. (2015) previously reported that CS-exposed mice infected with NTHi were associated with an induction of IL-17A in the lungs followed by neutrophil recruitment, reinforcing the strength of the inflammatory process mediated by Th17 cells in NTHi-associated AECOPD (37).

The comparison of previous studies using human lung samples (29, 42) with the lungs of CS-exposed mice shows that the main difference in the inflammatory signal is the lung compartment in which they are detected. It appears that in humans, the inflammatory infiltrate is mainly observed in the airways between the respiratory epithelium and the adventitia (15, 42, 65), whereas in a CS-exposed animal model, inflammatory infiltrates are observed mostly in peribronchovascular areas between the airways and adjacent blood vessels (25, 26, 35). This difference likely occurs due to the specialized capillaries in this area, which allow for the rapid release of leukocytes and therefore results in oedema and inflammatory infiltration (66).

## 4 Treatments Targeting Th17/Treg Cells in COPD and *In Vitro* Studies

The most common treatment of COPD patients is the use of long-acting β2-agonists and anticholinergic drugs, which improves the clinical manifestations of airway obstruction. Moreover, during exacerbations or depending on the COPD stage, the combination of anti-inflammatory treatment with inhaled corticosteroids (ICS) and one or two long-acting bronchodilators (1), is also used, which is known to reduce the number of CD8+ T lymphocytes in the bronchial mucosa of those patients (67). However, the anti-inflammatory effects on other adaptive and innate immune cells were much lower than those observed in asthma (67, 68). Thus, studies that attempt to identify new treatment targets are necessary to better understand the imbalance between pro- and anti-inflammatory responses in COPD. In this context, *in vitro* and experimental studies are useful (69).

Profita et al. (2014) demonstrated in PBMCs cultured cells that treatment with tiotropium spiriva, an anticholinergic drug, and olodaterol, a long-acting β2-agonist, can control systemic inflammation through the Th17/Treg balance (70). The authors found that T-cells from COPD patients had higher levels of the neurotransmitter of the cholinergic system acetylcholine (Ach) coexpression with IL-17A, IL-22 and RORγt and that ACh might promote the increased levels of Th17 cells in systemic inflammation. Additionally, treatment with tiotropium and olodaterol reduced the percentage of T cells coexpressing AChIL-17A, AChIL-22, and AChRORγt while increasing the Foxp3 expression in COPD patients (70).

Using antibiotics, Tan et al. (2016) evaluated treatment with erythromycin for 6 and 12 months in induced sputum and peripheral blood of COPD stage II-IV patients (71). The authors showed that the treatment was effective in decreasing the IL-17 and IL-23 levels in both the sputum and serum of individuals treated for 12 months compared with the placebo-treated group and 6-month-treated group (71).

In accordance, mice that received elastin peptides (EP) intranasally, treatment with erythromycin was sufficient to reduce the IL-17 and IL-6 levels in both the serum and BALF compared to the EP group (72). Additionally, treatment with erythromycin modulated the CD4+ T cell responses by reducing the Th17 cells (IL-17+CD4 T+ frequency and *IL-17* mRNA) and increasing the Treg response (FOXP3+CD4+ CD25+ frequency and *Foxp3* mRNA) in the peripheral blood, spleen, and lungs (72). Additionally, Liu et al. (2020) treated cigarette smoke extract (CSE)-exposed dendritic cells (DCs) with erythromycin and demonstrated that treatment suppressed the CSE-exposed DC-mediated polarization of CD4+ T cells into Th17 cells (73).

Treatment with N-Acetylcysteine (NAC), an antioxidant that works as a mucolytic agent, was also effective on restore the Th17/Treg imbalance in COPD patients (74). In this study, the oral administration of NAC for 6 months led to a decrease in Th17 cells and increase in Treg cells of PBMC from COPD treated patients compared to the nontreated group. These findings were accompanied with decreased serum levels of inflammatory cytokines such as IL-17 and IL-1β and increased of the anti-inflammatory IL-10 level (74). In addition, the authors demonstrated that NAC regulated Th17/Treg balance through Hypoxia Inducible Factor-1α (HIF-1α) pathway, which is molecule that can upregulate the IL-17 expression through RORγt and in addition to degrade Foxp3 (74, 75). The COPD NAC-treated group showed significantly decreased HIF1-α expression of Th17 and Treg cells isolated from peripheral blood (74).

More recently, the efficiency of treatment with anti-IL-17 has been demonstrated to improve the inflammation observed in asthma (76, 77), asthma exacerbated by LPS (78, 79) and LPS-induced lung injury (80). In an elastase-induced model of emphysema, it was also demonstrated that both preventive and therapeutic treatment with anti-IL17 improved the evaluated inflammatory parameters in mice, including specific markers for the Th17 response (81).

Considering the importance of corticosteroids, Nasreen et al. (2014) demonstrated that treatment with fluticasone furoate and mometasone furoate, two glucocorticoids usually recommended for the treatment of allergic rhinitis and other inflammatory diseases, in epithelial cell cultures exposed to cigarette smoke, promoted an increase in SOCS3 (82). Considering that SOCS3 can prevent Th17 skewing through STAT3 inhibition, the authors suggested a new mechanism for this drug to induce an anti-inflammatory effect. In addition, both treatments were able to restore the inhibited SOCS3 expression in the airway epithelium of CS-exposed mice (82). In contrast, treatment with budesonide (ICS) and formoterol (long-acting β2-agonist bronchodilator), both commonly used for COPD treatment, was ineffective in restoring the loss of CD4+CD25highCD127- cells, a Treg population with highly suppressive functions isolated from PBMCs of both current and former COPD smokers (83).

## 5 Final Considerations and Future Directions

It has been demonstrated that in both clinical and experimental studies, an increased Th17 response is involved in the progression of obstruction in COPD, even when comparing different lung compartments or systemic and local responses. These changes are frequently observed in more advanced stages of disease progression in experimental studies, while clinical studies demonstrate Th17 skewing since early COPD stages.

**Figure 1:**
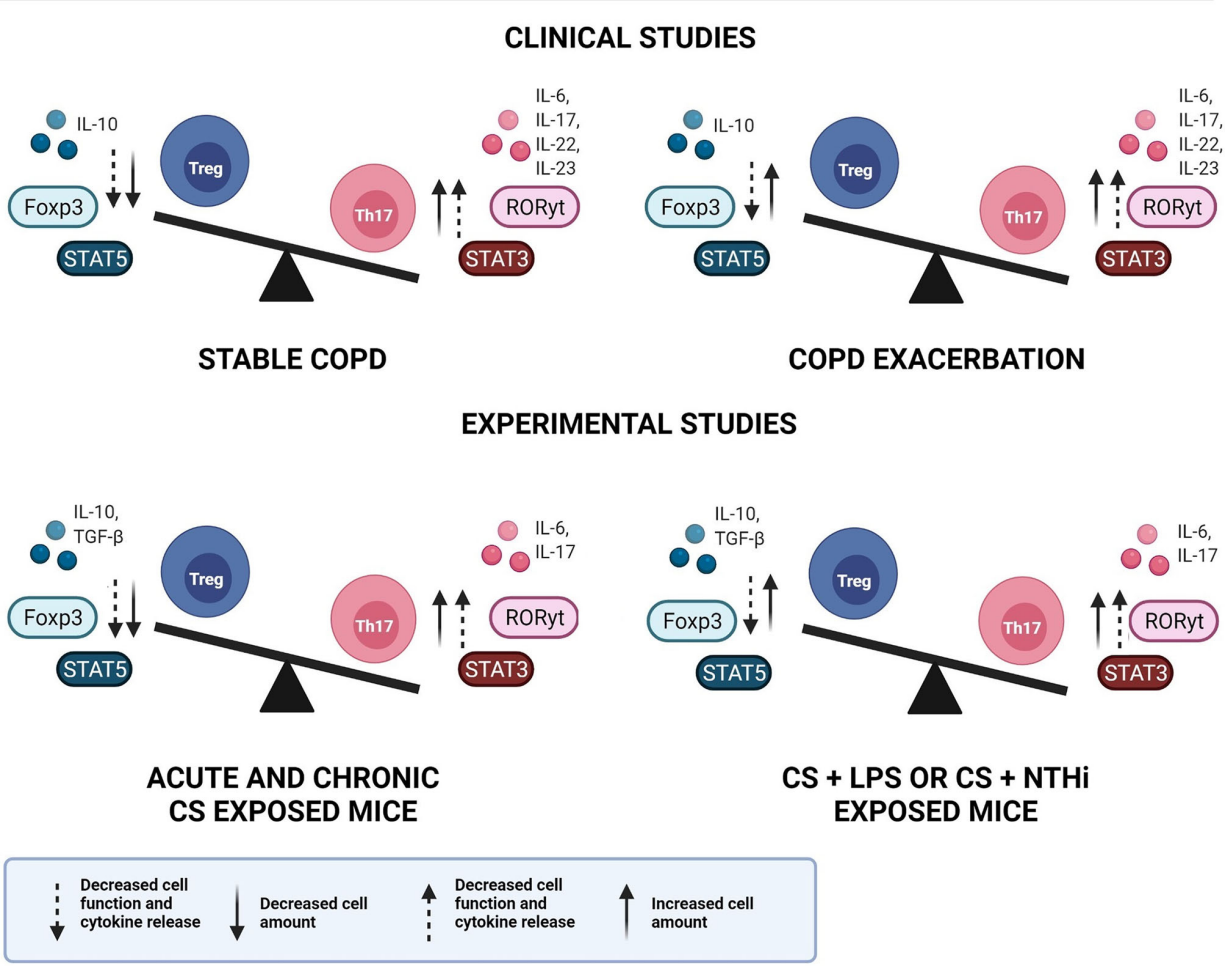
Th17/Treg imbalance in COPD. Representative image of Th17/Treg imbalance in clinical and experimental studies, in both stable and exacerbated disease. An increase in both Th17 cell amount and function is observed in clinical and experimental studies, during stable and exacerbated disease. In contrast, the Treg response may present increased cell amounts, however with decreased function and cytokine release, leading to Th17/Treg imbalance. COPD, chronic obstructive pulmonary disease; Treg, regulatory T cell; Th17, T helper-17, IL, interleukin; Foxp3, Forkhead box p3; STAT, signal transducer and activator of transcription; RORγt, retinoic acid orphan receptor; TGF-β, transforming growth factor-β; CS, cigarette smoke; LPS, lipopolysaccharide; NTHi, *Haemophilus influenzae*.

On the other hand, discrepant results have been observed, especially in studies that have described the immune response mediated by Treg cells. Depending on the compartment analysed, or if lung and blood samples are collected from the same individual, differences are observed in Treg amounts and/or function. Nevertheless, most studies are in agreement with the IL-10 analysis, describing the decrease in IL-10 release during COPD development and progression. **Figure 1** elucidates the general findings about Th17/Treg imbalance in clinical and experimental studies, in both stable and exacerbated disease, considering not only the cell amounts but also the cells function and the release of related cytokines.

Considering that Foxp3-expressing Treg cells are a heterogeneous cell group comprising distinct subpopulations with different phenotypes and functions (84), it is reasonable to think that to better understand the physiological mechanisms in COPD development, it is more important to distinguish these different cell phenotypes instead of the total number of Tregs. However, until now, there have been only a few studies describing these different phenotypes as well as the immunosuppressive activity of these cells in COPD.

There are 3 described subtypes of Treg cells: resting Tregs (rTregs, CD25++CD45RA+), activated Tregs (aTregs, CD25+++ CD45RA-) and cytokine-secreting Tregs (Frlll, CD25++ CD45RA-Frlll), which were demonstrated to be imbalanced in COPD. Resting and activated Treg cells have suppressor activity, while Treg Frlll cells have a proinflammatory capacity and can produce IL-2, IL-17 and interferon-γ (IFN-γ) (85).

Hou et al. (2013) evaluated the proportion of Treg cell phenotypes (rTregs, aTregs, and Treg-Frlll) in the peripheral blood and BALF of nonobstructive smokers, never-smokers and individuals with COPD in stages II and III (84). Blood samples demonstrated decreased rTreg and aTreg proportions and increased Treg-Frlll in COPD individuals compared to nonobstructive smokers. Similarly, decreased aTreg cells and increased Treg-FrIII levels were demonstrated in the BALF samples of COPD patients coto healthy smokers and never-smokers. In addition, the authors observed a decrease in the (aTreg + rTeg): Treg-Frlll ratio in both blood and BALF. These findings suggest that the imbalance between the anti-inflammatory subpopulations (aTreg + rTeg) and the proinflammatory subpopulation (Fr III) of Tregs plays an important role in the progression of COPD (84).

The imbalance between Treg cell subpopulations was also demonstrated in individuals with COPD after exacerbation. Yang et al. (2017) found an increase in the percentage of Treg cells secreting cytokines and a decrease in resting Treg cells in both patients with stable COPD and individuals with COPD after exacerbation (86). The authors suggested that there is an increased expression of Treg subtypes with low or no suppressive activity in the blood of individuals with COPD, accompanied by decreased expression of Treg subtypes with suppressive activity (86).

Since there is evidence of increased Treg cells with low or no suppressive activity in COPD individuals, additional studies will be necessary to investigate the distribution of these phenotypes in different stages of COPD. The elucidation of these cell functions as well as which physiological mechanisms orchestrated by these cells are involved in COPD progression seems to be essential to better elucidate this respiratory disease development and progression. In addition, this could be a promising pathway to find new therapeutic approaches.

## Author Contributions

FD conceived of the manuscript. JL and FD drafted the manuscript. JL and JI created figures and tables. JL, IT, and MM provided literature search and edited the manuscript. All authors were involved in scientific discussion of the review. All authors contributed to the manuscript and approved the submitted version.

## Funding

This research was supported by FAPESP (2019/25374-7).

## Conflict of Interest

The authors declare that the research was conducted in the absence of any commercial or financial relationships that could be construed as a potential conflict of interest.

## Publisher’s Note

All claims expressed in this article are solely those of the authors and do not necessarily represent those of their affiliated organizations, or those of the publisher, the editors and the reviewers. Any product that may be evaluated in this article, or claim that may be made by its manufacturer, is not guaranteed or endorsed by the publisher.
